# A First- and Second-Order Motion Energy Analysis of Peripheral Motion Illusions Leads to Further Evidence of “Feature Blur” in Peripheral Vision

**DOI:** 10.1371/journal.pone.0018719

**Published:** 2011-04-29

**Authors:** Arthur G. Shapiro, Emily J. Knight, Zhong-Lin Lu

**Affiliations:** 1 Department of Psychology, American University, Washington, D.C., United States of America; 2 Guggenheim 2 Mayo Graduate/Mayo Medical Schools, Rochester, Minnesota, United States of America; 3 Departments of Psychology and Biomedical Engineering, Neuroscience Graduate Program, University of Southern California, Los Angeles, California, United States of America; Duke University, United States of America

## Abstract

**Background:**

Anatomical and physiological differences between the central and peripheral visual systems are well documented. Recent findings have suggested that vision in the periphery is not just a scaled version of foveal vision, but rather is relatively poor at representing spatial and temporal phase and other visual features. Shapiro, Lu, Huang, Knight, and Ennis (2010) have recently examined a motion stimulus (the “curveball illusion”) in which the shift from foveal to peripheral viewing results in a dramatic spatial/temporal discontinuity. Here, we apply a similar analysis to a range of other spatial/temporal configurations that create perceptual conflict between foveal and peripheral vision.

**Methodology/Principal Findings:**

To elucidate how the differences between foveal and peripheral vision affect super-threshold vision, we created a series of complex visual displays that contain opposing sources of motion information. The displays (referred to as the peripheral escalator illusion, peripheral acceleration and deceleration illusions, rotating reversals illusion, and disappearing squares illusion) create dramatically different perceptions when viewed foveally versus peripherally. We compute the first-order and second-order directional motion energy available in the displays using a three-dimensional Fourier analysis in the (x, y, t) space. The peripheral escalator, acceleration and deceleration illusions and rotating reversals illusion all show a similar trend: in the fovea, the first-order motion energy and second-order motion energy can be perceptually separated from each other; in the periphery, the perception seems to correspond to a combination of the multiple sources of motion information. The disappearing squares illusion shows that the ability to assemble the features of Kanisza squares becomes slower in the periphery.

**Conclusions/Significance:**

The results lead us to hypothesize “feature blur” in the periphery (i.e., the peripheral visual system combines features that the foveal visual system can separate). Feature blur is of general importance because humans are frequently bringing the information in the periphery to the fovea and vice versa.

## Introduction

Anatomical and physiological differences between the foveal and peripheral visual systems are well documented. At the level of the retina, the fovea contains a higher ratio of cone to rod photoreceptors [1] and a higher density of ganglion cells [2]. The primate fovea (unlike other mammalian foveae) is disproportionately populated by midget retinal ganglion cells [3] that have a characteristic morphology unlike other regions of the retina (see [4]). In the primary visual cortex, the area that responds to signals originating in the fovea covers a disproportionately larger region than the area that responds to the retinal periphery [5] [6]. The anatomical projections from V1 to other cortical areas appear to differ dramatically depending on whether those projections originated in the central or peripheral regions of the cortex [7], and projections from non-visual extrastriate cortical areas to V1 seem to target the peripheral visual cortex but not the central visual cortex [8].

A longstanding question in vision science concerns how these anatomical and physiological differences between the fovea and the periphery affect our perception. One prominent hypothesis is that vision in the periphery is primarily a spatially, temporally, and photometrically scaled version of vision in the fovea. Such a view is supported by findings that grating sensitivity and Vernier acuity measured in the periphery match measurements in the fovea scaled by a factor that accounts for the differing distributions of ganglion cells (M scaling) [9] [10]. However, other findings suggest that vision in the periphery cannot be fully explained by the scaling of foveal vision. For instance, research on visual crowding, a phenomenon in which visual object recognition is impaired by the presence of visual clutter [11] [12], suggests that the fovea is better than the periphery at representing spatial and temporal phases [13] [14] [15] [16] [17], and that vision in the periphery has difficulty representing features at a stage beyond feature detection [18] [19] [20] [21] [22]. Cortical scaling also fails to account for recognition performance in complex stimuli [23] [24] and for foveal and peripheral differences in contrast suppression and contrast facilitation [25].

If the visual periphery is poor at representing spatial and temporal phases and other visual features, the perceptual ramifications should be dramatic at super-threshold levels. This appears to be the case. For instance, Shapiro, Lu, Huang, Knight, and Ennis [26] examined the curveball illusion, which juxtaposes two orthogonal motion signals: a global motion signal (a disk descends vertically from the top to the bottom of the screen); and a local motion signal (right-to-left motion of the stripes inside the disk). If an observer tracks the disk foveally, the disk appears to descend vertically; however, if an observer shifts his/her gaze to the right so that the disk falls in the far visual periphery, the disk appears to drift to the left at an oblique angle. The effect is a variation of other phenomena in the literature that suggest poor phase discrimination in the periphery [27] [28] [29] [30] [31] [32].

Another example that shows poor feature representation in the periphery is the contrast asynchrony phenomena [33] [34] [35]. The typical contrast asynchrony stimulus consists of two disks whose luminance levels modulate simultaneously in time; one disk is placed against a light background, and the other is placed against a dark background. The contrast asynchrony juxtaposes two sources of information: an in-phase modulation from the luminance of the disks, and an antiphase modulation that arises from the contrast between the disks and the surrounding background. When the contrast asynchrony stimulus is viewed foveally at 1 Hz, the visual system is able to separate the two sources of information, and creates the paradoxical perception that the disks modulate in antiphase but become light and dark at the same time. When viewing the contrast asynchrony stimulus peripherally, many observers report that they see the luminance information but not the antiphase contrast information. The disappearance of the antiphase percept is not due only to poor spatial resolution in the periphery because the contrast percept remains in the fovea even after considerable optical blur (see [35], Fig. 10). The disappearance of the antiphase percept in the periphery, therefore, is consistent with the hypothesis that the periphery is relatively poor at representing the temporal phase of the contrast modulation and therefore combines features that the foveal visual system can process separately. It is also possible that the second-order system has a diminished response in the periphery at lower spatial frequencies.

Here we examine the hypothesis (which we call “feature blur”) that the peripheral visual system combines features that the foveal visual system can separate. To do this, we use a series of super-threshold visual stimuli that generate dramatically different percepts (“illusions”) when viewed in the fovea versus in the periphery. Like the contrast asynchrony and the curveball illusion, the illusions presented here contain different sources of information that are in conflict with each other. We analyze these phenomena with the same three-dimensional Fourier analysis previously applied to analyze the curveball illusion. The analysis represents the motion in a three-dimensional space by projecting the (x, y, t) image cube on the x-t and y-t planes. To identify the second-order motion energy, we calculated the Michelson contrast of each point in each movie frame, removed the DC component in each frame by subtracting from the contrast images of each movie frame the mean x-y image of all the movie frames, and then applied a full-wave rectification to all of the resulting images.

The (x, y, t) space allows for a description of the complex illusions in terms of first-order directional motion energy and second-order directional motion energy [36] [37]. First-order directional motion energy refers to motion associated with objects or features that differ from the background in term of luminance. Second-order directional motion energy refers to motion or flicker in which the moving object is defined by the amount of visual feature (e.g., contrast) and there is no difference in mean luminance between target and background [38] [39]. The analysis of complex super-threshold motion phenomena is important because the human visual system regularly brings information from the periphery to the fovea and vice versa. The results provide additional support for the hypothesis that the peripheral visual system combines features that the foveal visual system can process separately.

## Methods

### 1. Demonstration programs

The demonstration programs for our super-threshold visual phenomena were created in Adobe Flash CS3 and were programmed in Actionscript 2, a scripting language that is built into the Flash programming environment.

### 2. Data Collection

We presented the displays in a classroom situation, to 26 American University students between the ages of 19 and 24. The size of the stimulus in terms of visual angle was dependent on the row in which students sat; students' chairs ranged from approximately 3.0 meters to 6.7 meters from the screen. The projected size of the image on the screen was 1.2×1.8 meters (i.e., observers in the front of the room saw a projection that was approximately 22×31 deg of visual angle, and observers in the back of the room saw a projection that was approximately 10×15 deg of visual angle). The display was controlled from a Macbook Pro connected to a Sanyo PLC XT25 theater projector.

The demonstrations have been presented to both small and large public audiences (for instance, at Vision Science Society's Demonstration Night and the Best Illusion of the Year contest in 2008 and 2009). The phenomenal differences between peripheral and foveal viewing are robust over a wide range of viewing configurations, distances, and scales. For the purposes of most demonstration programs (with the exception of the Kanisza illusion, whose procedure will be discussed below), we were interested in documenting the existence of the effect.

### Ethics Statement

The American University Institutional Review Board approved the experimental protocol for these experiments.

### Procedure

Student observers at American University were given a response form with questions concerning the displays. Students were informed orally and in writing (on the first page of the response form) about the conditions of the study, that participation in the study was anonymous and voluntary, that their responses would be part of a data set that may be published in scientific proceedings, that turning in a completed response form indicated their informed consent to be part of the study, that they could turn in a blank response form or hold on to the response form if they did not wish to participate, and that there was no penalty for not participating. The presentation of the trials corresponded to potential responses on the response form. The demonstrations were presented on the classroom projector system. After each demonstration was presented, the participants wrote down their responses; when all participants had finished recording their responses to a demonstration, the next demonstration was presented.

### 3. Motion Energy Analysis

**Figure 1:**
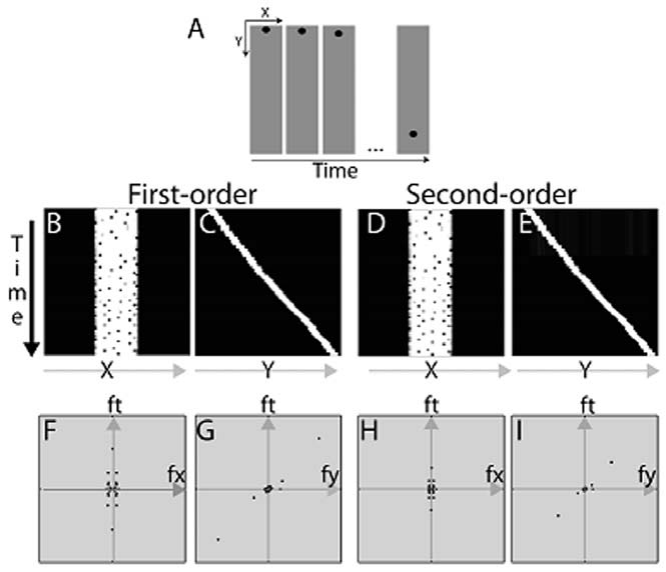
Motion energy analysis for a solid dropping disk. A) A series of frames depicting a solid dropping disk. B&C) First-order motion plots in the x-t and y-t planes. D&E) Projections of DC-removed and rectified second-order dropping disk movie in the x-t and y-t planes. F–I) Fourier analysis of the first-order and second-order motion energy of the solid dropping disk in the fx-ft and fy-ft planes.

The motion energy analysis was detailed in [26]. In brief, the analyses were performed on a series of still images (movie frames) created with the aid of a Flash-Video converter (MacVide). The method is illustrated with a dropping solid ball (Fig. 1A). For each illusion, we first computed the Michelson contrast of each point (x, y) in each movie frame and then placed all the images at different time points in the three-dimensional (x, y, t) space. For a dropping solid ball, projections of the resulting three-dimensional volume in the x-t and y-t planes are shown in Figures 1B and C.

To compute first-order motion energy in the horizontal and vertical directions, we first computed the Fourier power spectrum of the three-dimensional volume using Matlab 7.4. We then projected the three-dimensional Fourier power spectrum onto the fx-ft and fy-ft planes. In Figures 1F and G, polar plots of the Fourier power are summed over every 15 degs in the fx-ft and fy-ft planes, respectively. Note that the different directions in the fx-ft and fy-ft planes represent different speeds in the horizontal and vertical directions, respectively. For any point in the fx-ft and fy-ft planes, the larger the slope of the line connecting the point to the origin, the faster the motion. In both the fx-ft and fy-ft planes, we define motion energy, 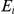, in a particular quadrant, i, as the sum of Fourier power in that part of the Fourier space. The total motion energy, whose sign determines the direction of motion, is defined as
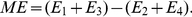
(1)In the fx-ft plane, positive motion energy signifies motion from the left to the right; negative motion energy signifies motion from the right to the left. In the fy-ft plane, positive motion energy signifies motion from the top to the bottom; negative motion energy signifies motion from the bottom to the top. In both planes, zero motion energy signifies no motion.

To take into account contrast-gain control in motion systems [40], a normalized measure of motion energy,
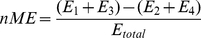
(2)was computed and used to estimate the presence or absence of horizontal and vertical motion in a display. In Eq. 2, *E_total_* is the total Fourier energy in the fx-ft plane or the fy-ft plane. *E_total_* includes energy in the four quadrants and on the axes. For the motion stimuli in Figure 1, nMEx = 0.00, and nMEy = 0.75, reflecting no motion in the horizontal direction but significant top-to-bottom motion in the vertical direction.

To compute second-order motion energy, we computed the Michelson contrast of each point in each movie frame, removed the DC component in each frame by subtracting from the contrast images of each movie frame the mean x–y image of all the movie frames, applied a full-wave rectification (square) on each point of all the resulting images [38] [39] and placed all the resulting images at different time points in the three-dimensional (x, y, t) space. For the dropping solid ball in Figure 1, projections of the resulting three-dimensional volume in the x-t and y-t planes are shown in Figures 1D & E. The remaining steps of the analysis are identical to those in the first-order analysis. For the stimuli in Figure 1, the normalized second-order motion energy in the horizontal direction is nMEx = 0.00, signifying no motion in the horizontal direction. The normalized second-order motion energy in the vertical direction is nMEy = 0.74. Therefore, consistent top-to-bottom motion energy is present in the first- and second-order motion systems.

## Results

### Demonstration 1: Peripheral escalator illusion

Object identification depends upon the visual system's ability to distinguish between individual features and then select and bind features into a group. Such a task seems to require knowledge of the relationship of the object relative to the background. For instance, to identify an object whose border is partially occluded, the observer must be able to line up the individual line segments, and if the object is moving relative to the background, the observer must be able to organize features based on the synchrony of the movement (consider examples of the Gestalt principle of common fate). If the peripheral visual system combines features under some conditions, then there should be conditions in which objects that are well defined for foveal vision become poorly defined in the periphery.

**Figure 2:**
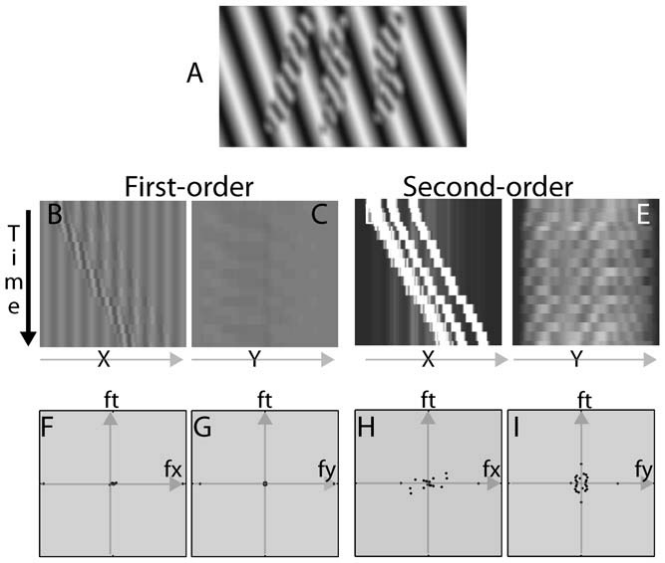
Motion energy analysis for the peripheral escalator illusion (See Movie S1). A) A single frame of the peripheral escalator illusion. The background is a stationary gradient. The three blurred columns shift horizontally back and forth. The columns are perceived as drifting horizontally when viewed in the fovea, but obliquely when viewed in the periphery. B&C) First-order motion plots in the x-t and y-t planes. D&E) Projections of DC-removed and rectified second-order peripheral escalator movie in the x-t and y-t planes. F–I) Fourier analysis of the first-order and second-order motion energy of the peripheral escalator movie in the fx-ft and fy-ft planes.

To test the (in)ability of peripheral vision to segregate features, we created a configuration of three striped columns that drift horizontally back and forth across the screen in front of a grating background tilted at 45 degrees (Fig. 2A, Movie S1). The configuration pits two types of features against each other: 1) if the visual system perceives the columns as objects, then the columns should separate from the background and appear to move horizontally; 2) if the visual system does not perceive the columns as objects, then motion should arise from the oblique intersections of the columns and the background gratings – something akin to a barber-pole illusion. The image therefore allows for two interpretations: if an observer is able to separate the columns from the background, then he or she should see columns of columns drift across the grating background; if, however, the observer cannot discern the columns as individual objects, the intersections between a column and the background become more salient (as would be the case if there is poor feature binding or poor phase representation). In this situation, the observer should see the motion drift in the direction of the intersections. This type of effect has precedents in the literature [27], in which a moving single line against a grating appears to drift at different angles as the line moves into the periphery.

The effect of this pattern can be seen in Movie S1. If an observer fixates on the drifting columns (thereby placing the image of the drifting columns in the fovea), the columns are easily separated from the background and appear to drift back and forth horizontally. If the observer looks several inches above the display (thereby placing the image of the drifting columns in the far periphery), the columns shift direction and appear to move obliquely at about 45 deg. The effect is not due simply to blur, since a blurring of the display with a handheld lens leads to the perception of vertical internal motion in foveal vision. Therefore, it seems as if the peripheral percept occurs because the periphery confounds the horizontal motion of the columns with the vertical motion signals contained in the low spatial frequencies of the images.

#### Documentation of the effect in a classroom setting

Movie S1 was projected in the classroom setting. The striped columns were projected at a size of about 7×23 cm (i.e., the columns were approximately 1.3×4.4 deg of visual angle for observers in the front of the room, and 0.6×2.0 deg of visual angle for observers in the back of the room), and the background grating had a size of 0.3×1.8 meters (5.7×31 deg in the front and 2.6×15 deg in the back). Observers saw two different conditions of the peripheral escalator illusion: one with a narrow background grating (i.e., the width of columns) and one with a wide background grating (i.e., about 2.5 times the width of the columns). The width of the grating was determined by the size of the background bars in pixels (a size of 40 pixels for the narrow width, and a size of 100 pixels for the wide width).

When viewing in the periphery, 69% percent of the participants reported seeing the columns drift upwards when the columns were viewed against the narrow bars, whereas 0% reported seeing the columns drift upwards against the wide bars. The 69% value may seem low, but it is not surprising given the observers' different distances from the screen.

#### First-order Analysis

Projections of the peripheral escalator movie in the x-t and y-t planes are shown in Figures 2B and C. As can be seen from the figures, there is not much consistent first-order slant in the x-t plane and no dominant slant in the y-t plane. Fourier analysis confirmed the observation. In Figures 2F and G, most of the motion energy is on the axes, nMEx = 0.007, and nMEy = 0.005.

We used a form of contrast gain control derived from pedestal experiments, in which the pedestals and motion stimuli were shown very briefly [40]. In the peripheral escalator illusion, the background sine wave pattern is shown to be continually present. The small normalized motion energy represents perhaps a much lower estimate of the motion energy in the first-order system if adaptation is taken into account. The important point here is that the amount of motion energy in the horizontal and the vertical directions is almost the same – when combined, they result in the peripheral escalator illusion.

#### Second-order Analysis

Projections of the DC-removed and rectified second-order escalator movie in the x-t and y-t planes are shown in Figures 2D and E. As can be seen from the figures, there is a significant second-order slant in the x-t plane; there is also a small second-order slant in the y-t plane. Fourier analysis found that nMEx = 0.201, and nMEy = 0.049. In Figures 2H and I, the positive motion energy in both the fy-ft and fx-ft planes signifies the presence of motion energy in both the left-to-right and the top-to-bottom directions.

#### Summary

One interpretation of the results is that the escalator motion in the periphery reflects integration of the horizontal and the vertical motion signals in the second-order system, and perhaps also in the first-order system. For example, consider the combination of first- and second-order motion. In the Fourier plots, there is significant left-to-right and top-to-bottom motion energy in the second-order motion system, but no significant motion energy in the first-order motion system. That may be due to an overestimate of contrast-gain control. If we discount contrast-gain control, there would be significant left-to-right and top-to-bottom motion energy in the first-order motion system. This interpretation suggests that foveal processing is able to maintain two separate representations (first order/second order or high spatial frequency/low spatial frequency), but the peripheral system cannot. It does not seem to be the case that feature blur represents a failure to segregate the field into objects (and so the features remain unattached), since in the periphery it is still possible to perceptually separate the objects from the background.

### Demonstrations 2 and 3: Peripheral acceleration and deceleration illusions

**Figure 3:**
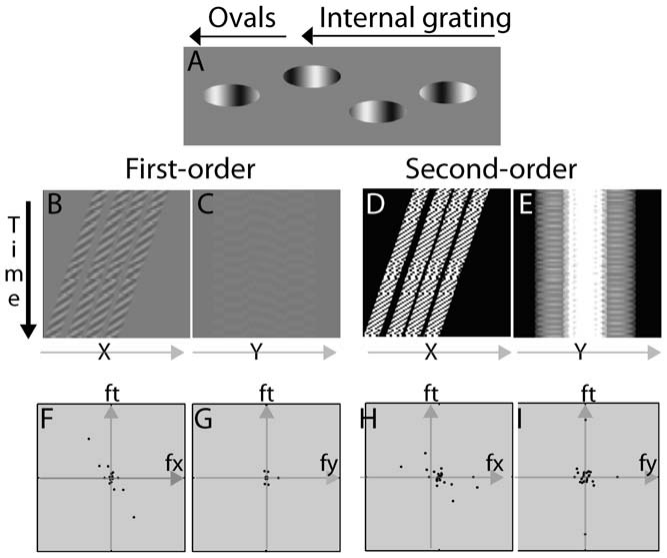
Motion energy analysis for the peripheral acceleration illusion (See Movies S2). A) A single frame of the peripheral acceleration illusion. Ovals drift from left to right across the screen. Inside each oval is an internal gradient that moves faster than the oval and in the same direction as the oval. When viewed foveally, observers can separate the ovals and internal grating; when viewed peripherally, the ovals appear to accelerate, and the interior of the oval appears fixed. B&C) First-order motion plots in the x-t and y-t planes. D&E) Projections of DC-removed and rectified second-order peripheral acceleration movie in the x-t and y-t planes. F–I) Fourier analysis of the first-order and second-order motion energy of the peripheral acceleration movie in the fx-ft and fy-ft planes.

**Figure 4:**
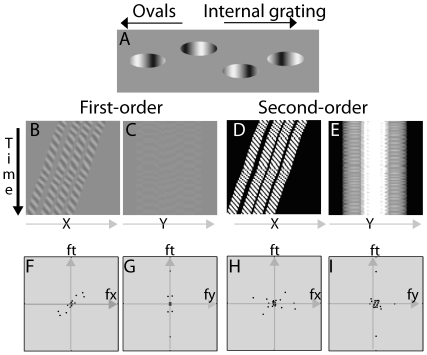
Motion energy analysis for the peripheral deceleration illusion (See Movie S3). A) A single frame in the peripheral deceleration illusion. Ovals drift from left to right across the screen. Inside each oval is an internal gradient that moves in the direction opposite to the motion of the oval. When viewed foveally, observers can separate the ovals and internal grating; when viewed peripherally, the speed of the ovals is determined by the internal motion, yet it is difficult to see the motion of the internal gradient. In the supplementary movie, the observer can adjust the speed of the internal grating. When the gradient is faster than the ovals, a shift from foveal to peripheral viewing will make the ovals appear to accelerate; when the gradient is slower than the ovals, a shift from foveal to peripheral viewing will make the ovals appear to decelerate and even reverse direction (creating the paradoxical view that the ovals are moving slowly leftward, yet somehow get to the far right side of the screen). B&C) First-order motion plots in the x-t and y-t planes. D&E) Projections of DC-removed and rectified second-order peripheral deceleration movie in the x-t and y-t planes. F–I) Fourier analysis of the first-order and second-order motion energy of the peripheral deceleration movie in the fx-ft and fy-ft planes.

The peripheral acceleration and deceleration illusions consist of ovals that drift from left to right across the screen, and that contain an internal grating moving in the same (acceleration) or opposite (deceleration) direction, and at a faster or slower speed than the motion of the ovals across the screen (see Figs. 3A and 4A; Movies S2 and S3). The controls in the demonstration program can be used to adjust the speed and direction of the internal grating. A similar illusion was created by Zhang, Yeh, and De Valois, who examined the direction of motion for a grating that moved behind a drifting aperture, and by Brady and Movshon [41] to study the relationship between the response of MT cells and neural correlates of consciousness.

The peripheral acceleration illusion (Movies S2) occurs when the motion inside the ovals is faster than the motion of the ovals but in the same direction. When the observer looks directly at the ovals, the observer can easily identify both the internal motion and the motion of the ovals. Two dramatic perceptual changes occur when the observer views the display in the periphery: first, the ovals appear to increase speed (i.e., they seem to take on the speed of the internal grating); and second, the internal grating appears to stop moving.

The perceptual deceleration illusion (Movie S3) occurs when the internal motion and the ovals move in opposite directions. When the display is viewed foveally, the internal motion can be separated from the motion of the ovals, but when viewed peripherally, the internal motion appears to stop, and ovals that are moving from right to left appear to move from left to right (i.e., the ovals appear to assume the direction of the internal grating). The effect when viewed peripherally is particularly paradoxical because even though the ovals appear to move from left to right, an observer can still detect that the ovals are reaching the left side of the screen.

#### First-order Analysis of Peripheral Acceleration

Projections of the peripheral acceleration movie in the x-t and y-t planes are shown in Figures 3B and C. Two significant slants in the x-t plane can be seen: the four stripes and their internal patterns, both in the same upper-right to lower-left orientation. No dominant slant appears in the y-t plane. These observations are confirmed by Fourier analysis (Figs. 3F and G): nMEx = −0.673, nMEy = −0.007. The negative motion energy in the horizontal direction signifies right-to-left motion. The near zero motion energy in the vertical direction signifies no motion in that direction.

#### Second-order Analysis of Peripheral Acceleration

Projections of the DC-removed and rectified second-order perceptual acceleration movie in the x-t and y-t planes are shown in Figures 3D and E. The figures have properties similar to those of Figures 3B and C. Fourier analysis (Figs. 3H and I) found that nMEx = −0.530, and nMEy = −0.031. The negative motion energy in the horizontal direction signifies right-to-left motion. The near zero motion energy in the vertical direction signifies no motion in that direction.

#### First-order Analysis of Peripheral Deceleration

Projections of the peripheral deceleration movie in the x-t and y-t planes are shown in Figures 4B and C. Two significant but opposite slants can be seen in the x-t plane, the four stripes in the upper-right to lower-left orientation, and their internal patterns in the upper-left to lower-right orientation. No dominant slant occurs in the y-t plane. Fourier analysis in the x-t plane computes the difference between the motion energies represented by the two different slants in the x-t plane (Figs. 4F and G). As a result, nMEx = 0.816, nMEy = 0.006. The positive motion energy in the horizontal direction signifies left-to-right motion, which is opposite to the direction of motion of the oval object. The near zero motion energy in the vertical direction signifies no motion in that direction.

#### Second-order Analysis of Peripheral Deceleration

Projections of the DC-removed and rectified second-order deceleration movie in the x-t and y-t planes are shown in Figures 4D and E. The figures have properties similar to those of Figures 4B and C. However, the energy of the slant of the stripes dominated that of the internal patterns. Fourier analysis (Figs. 4H and I) found that nMEx = −0.102, and nMEy = −0.032. The negative motion energy in the horizontal direction signifies right-to-left motion. The near zero motion energy in the vertical direction signifies no motion.

#### Documentation of the effect in a classroom setting

Observers in the classroom setting saw demonstration movies similar to Movies S2 and S3. In the classroom demonstration, ovals pass in front of a drifting grating; the grating therefore appeared as internal motion of the oval shapes that moved across the screen. For the classroom experiment, the ovals were projected at a size of approximately 5×23 cm (the columns were approximately 0.95×3.0 deg of visual angle for observers in the front of the room and .42×1.35 deg of visual angle for observers in the back of the room). Against a static grating, each oval masked 1.15 cycles of the internal grating and drifted at a rate of 1.1 cycles of the internal grating per sec. We controlled the speed of the grating by shifting the pattern by a fixed number of pixels on each frame. Participants saw three different speeds, corresponding to a shift of 14 pixels to the left (acceleration), −3 pixels to the right (near stop) and −8 pixels to the right (deceleration). The three different speeds corresponded roughly to 1.96 cycles/sec (acceleration), −.4 cycles/sec (near stop), and −1.0 cycles/sec (deceleration).

The participants reported whether the speed of the ovals in the periphery relative to the fovea sped up, slowed down, or remained the same. Most of the participants reported seeing the ovals speed up or slow down in all three conditions. The percentage of the class that did not see the effect (i.e., the percentage that reported that the ovals remained the same speed) was as follows: 19% for acceleration; 27% for near stop; 19% for deceleration. The effect was therefore seen by most of the group even when the difference of the internal motion was very small.

#### Summary

The peripheral acceleration and deceleration illusions are consistent with the hypothesis that the blurring of multiple sources of motion information occurs in peripheral vision. In the peripheral acceleration illusion, both the first- and the second-order systems have significant motion energy in the right-to-left (forward) motion direction. The perceived forward motion in the periphery reflects integration of the horizontal motion signals in both the first- and the second-order motion systems. In the peripheral deceleration illusion, the first-order system has significant motion energy in the left-to-right (backward) direction. The second-order system has significant motion energy in the right-to-left (forward) direction. The perceived backward motion in the periphery reflects integration of the horizontal motion signals in both the first- and second-order motion systems, where the stronger signal in the first-order system predominates.

### Demonstration Program 4: Rotational reversals

A limitation of three-dimensional Fourier analysis is that if the object moves in a circular path, the results of the analysis become uninterpretable. Here, we present a variation of rotational effects that highlights the dependence of peripheral illusions on contrast with the background (peripheral reversal illusion). These rotational illusions illustrate the effects of contrast level and temporal range of feature integration in peripheral vision.

**Figure 5:**
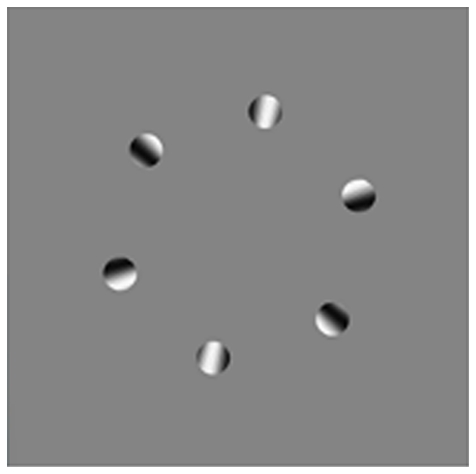
The rotational reveals illusion (see Movies S4). A ring of ovals moves counter-clockwise. Within each oval is an internal gradient that moves clockwise. When viewed foveally, the ring appears to rotate counter-clockwise, but when viewed peripherally, the ring appears to rotate clockwise. When the background is near the same luminance as the white or black of the internal gradient, the ring appears jumbled when viewed peripherally.

In the Rotational reversal illusion (Fig. 5 and Movies S4), six disks form a ring that rotates counter-clockwise while a grating inside each disk rotates clockwise. The internal grating was created by placing a large clockwise-rotating radial sine wave behind the six clockwise-moving disks. When viewed foveally, the ring appears to move counter-clockwise (following the actual motion of the disks), but when viewed peripherally, the ring appears to rotate clockwise, in line with the internal motion of the individual disks. This illusion was presented independently at the Society of Neuroscience conference by Meilstrup and Shadlen [42] and Shapiro, Knight, and Lu [43].

The difference between foveal and peripheral perceptions depends on the luminance of the background relative to the internal grating. Movies S4 allows the observer to adjust background luminance; when the background is white or black, the direction of motion in the periphery is not the reverse of the direction of motion in the fovea (i.e., the disks rotate counter-clockwise when viewed foveally and peripherally). On the small display, when the contrast is nearly equal to the maximum or minimum of the background, the motion of the disks becomes scrambled, as if the phase relationship is lost. Our interpretation of these effects is that the relative luminance alters the strength of second-order motion (the motion inside the disk) relative to first-order motion.

#### Documentation of the effect in a classroom setting

In the classroom presentation, the results were similar but not entirely in line with the observations on a computer monitor in the laboratory. In the projected display, each disk's diameter was approximately 3.8 cm, and covered approximately 1 cycle of the background grating. The ring (rotating counter-clockwise) made one complete rotation in approximately six seconds. The background disk that created clockwise internal motion consisted of 20 cycles of a radial sine wave and made one complete rotation in 8.3 seconds. Classroom participants viewed the rotating reversals display in the fovea and in the periphery at five different background levels (pixel values: 20, 65, 128, 195, and 255). The pixel value of 20 made the background darker than the internal rotation grating; values of 65 and 195 made the background near the minimum/maximum of the internal rotation grating; 128 made the background intermediate to the internal rotation grating, and 255 made the background brighter than the internal rotation grating.

Observers were asked whether the ring appeared to be rotating counter-clockwise or clockwise, or appeared to remain stationary, or appeared jumbled. The results from the classroom presentation are shown in table 1. When the display was viewed in the fovea, nearly all participants reported seeing the disks rotate counter-clockwise. When the display was viewed in the periphery, observers primarily reported seeing reversals (i.e., they saw the ring rotate clockwise—the opposite of the direction of foveal perception) when the background was in the mid-levels (i.e., pixel value 65, 128, and 195); observers did not report seeing reversals when the background was white and black (i.e., pixel value 20 and 255). Curiously, in the classroom situation, the ring appeared jumbled for about half the observers when viewed peripherally in the 20 and 255 background conditions. In the laboratory, most observers report that the disks appear jumbled in the 65 and 195 background conditions. The difference between these two presentation conditions may indicate differences in contrast and spatial scale, and clearly requires further investigation.

**Table 1:** Observer reports of the rotating reversals display in the fovea and in the periphery at five different background levels (pixel values: 20, 65, 128, 195, and 255).

N = 26	% Rotatingcounter-clockwise		% Rotatingclockwise		% Stationary		% Jumbled	
	Fovea	Periphery	Fovea	Periphery	Fovea	Periphery	Fovea	Periphery
20	26	15	0	1	0	0	0	10
65	25	0	0	26	0	0	1	0
128	23	0	0	25	0	1	3	0
195	25	2	0	24	0	0	1	0
255	26	11	0	0	0	3	0	12

The pixel value of 20 made the background darker than the internal rotation grating; values of 65 and 195 made the background near the minimum/maximum of the internal rotation grating; 128 made the background intermediate to the internal rotation grating; and 255 made the background brighter than the internal rotation grating. Observers were asked whether the ring appeared to be rotating counter-clockwise or clockwise, or appeared to remain stationary, or appeared jumbled.

The rotational movement is not easily analyzable by the three-dimensional Fourier analysis. The rotating reversals illusion demonstrates the effect of contrast on the perceptual resolution of conflicting sources of global and local motion information: when the background is gray, the direction of rotation depends on whether the display is viewed in the fovea or periphery; when the background is black or white, the direction of rotation does not depend on foveal or peripheral viewing; and when the background is nearly equal to the maximum or minimum of the internal grating, the display takes on a scintillating quality, and the direction of motion cannot be determined. The phenomenology is consistent with a hypothesis in which there are multiple responses to the stimulus (low- and high-frequency content, first- and second-order motion energy), and the relationship between the luminance of the background and the internal grating determines which combination of these responses will determine the ultimate perception. The scintillating pattern seems to represent a condition in which the responses are in relative balance, so no process has the dominant signal.

### Demonstration 5: The Disappearing Squares Illusion

Here we present another type of rotational display that demonstrates that the periphery groups or separates features more slowly than the fovea does. In Kanizsa figures, individual elements (often referred to as “pacmen”) contribute to give the impression of an illusory object. The visual system therefore selects from among possible interpretations: either there is a collection of individual elements, or there is a global feature constructed from these elements.

**Figure 6:**
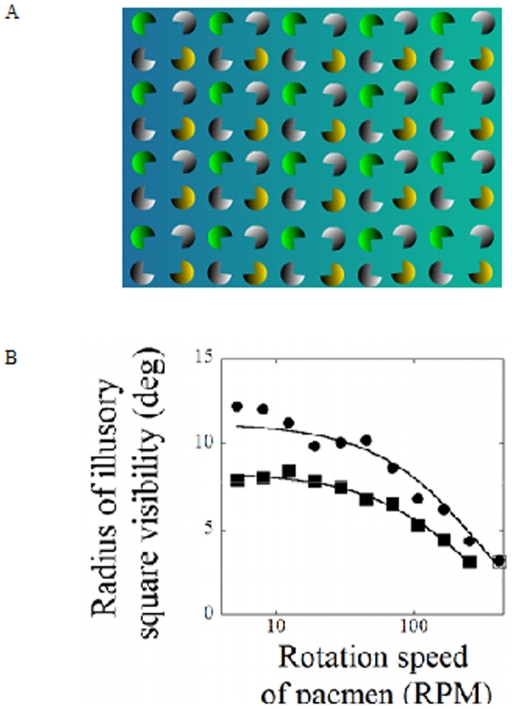
The Disappearing Squares illusion (see Movies S5). A) A 16×12 array of Kanizsa pacmen rotate in opposite directions so as to continually assemble/disassemble arrays of Kanizsa squares. B) Two observers adjusted the radius of the circles to encompass the range of visible squares as a function of rotation rate of the pacmen. The results for each observer are indicated by the squares and filled circles. As the rotation rate increases, the peripheral range over which the illusory squares can be seen decreases.

The spinning Kanizsa display examines the trade-off between these features as a function of the rate at which the elements spin. The spinning Kanizsa display (Figure 6, Movies S5) is a 16×12 array of Kanizsa squares. The squares, “pac-men” of different colors, are shaded so that the illusory segments are defined by the contrast with the background (in a manner similar to the illusory triangles of [44] and [45]). At first viewing, the display seems to argue against the “poor-phase” hypothesis. If the observer fixates in the center of the display, the 16×12 array of illusory squares can be seen throughout the periphery, whether the background is shaded or uniform gray and even with substantial stimulus perturbations to the background. In Movies S5, click on the “add/remove drifting background” button to place a drifting grating behind the pac-men. With the grating drifting in the background, the pac-men appear to bob up and down (the effect is similar to a “footsteps illusion,” only in two dimensions; see [46]), but the perception of illusory squares persists.

The illusory squares disappear in the periphery if the pac-men are rotated so as to continually assemble/disassemble arrays of Kanizsa squares (Figure 6A). As the rotation rate increases, the peripheral range over which the squares can be seen decreases, until, at fast rotations, the squares appear instantly at the point at which the observer fixates, but not at all in the periphery.

The spinning Kanizsa effect differs from the other demonstrations because it requires only foveal fixation, whereas the other demonstrations require a comparison of foveal to peripheral fixation; and because the phenomenon is one of extent (the perceived Kanizsa squares cover less of a range) rather than a qualitative change in appearance. We have therefore collected parametric data to document this effect.

#### Procedure

The stimuli for the experiment were presented on a Dell Optiplex Gx260 21″ monitor. The video driver was an Nvidia GeForce 6200 with 128 MB of memory. Calibration and gamma correction were checked using the onboard calibration system, a Cambridge Research Systems Optical photometer, and a Photoresearch Spectrascan 650 spectroradiometer. Observers viewed the stimuli from a distance of 54 centimeters using a chin rest for stabilization. The screen was 1280 pixels by 1024 pixels.

Adobe Flash was used to generate the stimulus for the experiment. Flash by itself does not allow data to be written to a disk. To overcome this difficulty, the Flash file was embedded in a Flash Projector (Zinc 3 produced by MDM) that turns Flash files into executable files. A photocell and oscilloscope were used to examine the waveform of the modulating lights produced by the executable file and produced no observable non-linearities or update interruptions when the executable file was running.

Two experienced psychophysical observers participated in this experiment: observer 1 was one of the authors (EJK); observer 2 was an undergraduate unaffiliated with the study.

We measured the range over which the illusory squares could be perceived as a function of the rotation speed. The display was similar to the display in Movies S5 except that four more pac-men were added to create a square 16×16 display. At the beginning of each trial, a 5.5 deg white ring was placed in the center of the display. The observer fixated on a dot in the center of the screen and used a computer mouse (click and drag motion) to adjust the radius of the ring so that the ring encompassed the range of the visible Kanizsa squares. After making the adjustment, the observer clicked on a control button, and the next trial began. There were ten rotation frequencies, and each frequency was presented four times in random order.

#### Results

In Figure 6B, the data are plotted as the radius of illusory-square visibility versus rotation rate and are shown for the two observers. The observers' data can be fit with exponential functions of radius versus rotation speed (for observer 1, top curve, the equation of the fitted line is: y = 11.22*exp(−0.0033*x); and for observer 2, the equation is y = 8.33*exp(−0.0039*x)). The two observers differed in the mean radius over which they perceived the Kanizsa squares; however, both observers showed a similar decrease in radius as a function of rotation speed.

#### Summary

The results from the spinning Kanizsa experiment are consistent with the perception that most viewers report when viewing Movies S5: as the speed of rotation increases, the range over which the Kanizsa squares can be perceived becomes narrower. At higher rates of rotation, the squares can only be perceived in the central 2 degrees. While the illusory squares can be seen in the periphery when the display is still, the addition of motion affects the periphery more than it does the fovea. The results are consistent with the hypothesis that as the rotation rate becomes faster, the periphery is relatively poor at combining features to create visual objects. While the peripheral visual system seems capable of creating objects from separate features, the ability to do this is constrained at some stage of visual processing, such as would occur if the processes required to create an illusory square have different temporal characteristics in the periphery and in the fovea.

## Discussion

We have presented a series of motion displays that demonstrate dramatic differences between central and peripheral vision. The primary question when examining differences in central and peripheral vision is whether the perceptual phenomena are simply the result of different cortical magnification factors. One way to test this hypothesis is to compare blurred versions of the visual displays viewed centrally to non-blurred versions of the display viewed peripherally. If peripheral vision is simply a low-pass version of central vision, then we should be able to simulate the effects in the periphery by removing the high spatial frequency content and viewing the display centrally. As we mention in the introduction, the blurred versions of the displays viewed centrally rarely produce an effect that is qualitatively similar to the non-blurred versions viewed in the periphery. However, such demonstrations cannot conclusively rule out the hypothesis that vision in the periphery is a scaled version of vision in the fovea because blurring a display cannot capture temporal differences between fovea and periphery.

The three-dimensional Fourier analysis of these displays demonstrates another way of interpreting differences between foveal and peripheral vision: in the fovea, the first-order motion energy and second-order motion energy could be separated from each other; in the periphery, the perception seems to correspond to a combination of the two sources of motion information. The peripheral combining of first- and second-order motion is similar to the hypothesis that the peripheral visual system combines multiple features into a single, integrated representation, leading to “abnormal integration at a stage beyond feature detection” [11], which has been proposed to account for (1) crowding phenomena [18] [19] [20] [21] [22]; (2) the misattribution of local motion signals to global objects in the infinite regress illusion [31]; (3) the integration of visual paths in the periphery [47] [48]; and (4) color disappearance in the periphery [49]. The hypothesis is also similar to the suggestion that the visual periphery has a reduced perceptual dimensionality relative to central vision [50].

We suggest the name “feature blur” for the hypothesis that peripheral vision combines first- and second-order motion processes because we speculate that the processes are part of a more general finding that the foveal visual system can maintain separate simultaneous representations of multiple features, but the periphery must somehow combine (or blur together) separate features into a more unified representation. The feature blur hypothesis stems partly from the 3-dimensional Fourier analysis and partly from the super-threshold phenomenology. The displays contain a number of features: first-order motion, second-order motion, motion direction, orientation, position, etc. If these features degraded separately, then we would expect to perceive a degraded, distorted jumble that would be identifiable—similar to the scintillation in the rotating reversal illusion when the background luminance is near the maximum or minimum luminance of the internal grating. However, when the features are clearly visible in the periphery (i.e., at levels above discrimination threshold), the perception does not correspond to a jumbled mixture of features: when viewing the peripheral acceleration (Fig. 3; Movies S2) and deceleration (Fig. 4; Movie S3) illusions peripherally, observers perceived ovals (the global feature) that accelerate or decelerate depending on the motion of the internal grating; when viewing the peripheral escalator illusion peripherally, observers perceived a row of ovals—objects—that move obliquely; and when viewing the falling ball illusion, shifting the gaze from the fovea to the periphery produces a perceived change in the ball's position, but observers still see the ball itself as a visual object [26]. The peripheral perception therefore seems to correspond to a single representation with a separate weighting for each of the features.

A combination of first- and second-order motion energy is not necessarily what one would expect to find in the periphery. For instance, the measurements may indicate a response only to first-order information or only to second-order motion information. Previous studies have shown that sensitivity loss for first-order motion is similar to sensitivity loss for second-order motion as a function of stimulus eccentricity [51] [52] but this does not seem to be true for all tasks [53]. Bressler and Whitney [32] have shown distinct position assignment mechanisms for first- and second-order motion. They directly measured position shifts produced by first- and second-order motion at 10.7 degree eccentricity and found that first-order motion influences position assignment across a broad range of temporal and spatial frequencies, and second-order motion influences perceived position over a narrower range of temporal frequencies and is largely invariant with spatial frequency. Bressler and Whitney's results indicate that near discrimination threshold levels, the location assigned to an object depends on multiple motion pathways, and may occur at multiple stages. From our analysis of super-threshold images, when both first- and second-order motion processes are in operation, the perceived position of an object (or direction of motion) when viewed peripherally is determined by a combination of motion responses. It therefore seems likely that feature combination occurs relatively late in the processing stream (i.e., after first- and second-order motion extraction) and that the weighting of the feature combination differs dramatically as a function of eccentricity.

### Summary and Conclusion

We created a series of visual displays (“illusions”) that emphasize differences between foveal and peripheral processing. The principle behind the displays is the juxtaposition of multiple sources of information so that there will be different foveal and peripheral perceptual interpretations that depend on what information the visual system extracts from the environment (similar in principle to [33] [35]). We examined the first- and second-order motion energy content in the displays by means of a novel three-dimensional Fourier decomposition. A comparison between the analysis and the perception of the displays suggests that the foveal visual system is capable of maintaining separate representations of first-order motion energy and second-order motion energy, but the peripheral visual system seems to combine the two sources of motion information. Based on the phenomenology of the displays, we contend that the inability of the peripheral visual system to separate first- and second-order motion energy is part of a general process in which the peripheral visual system blends together multiple features – a concept we refer to as “feature blur.” Because the effects are so dramatic and so easy to produce at super-threshold levels, we hypothesize that examples of feature blur may arise frequently in the natural environment whenever objects with conflicting sources of information undergo a transition between peripheral and foveal viewing.

## Supporting Information

Movie S1 **Peripheral escalator illusion.** The background is a stationary gradient. The three blurred columns shift horizontally back and forth. The columns are perceived as drifting horizontally when viewed in the fovea, but obliquely when viewed in the periphery.
(SWF)

Movie S2 **Peripheral acceleration illusion.** Ovals drift from left to right across the screen. Inside each oval is an internal gradient that moves faster than the oval. When viewed foveally, observers can separate the ovals and internal grating; when viewed peripherally, the ovals appear to accelerate, and the interior of the oval appears fixed.
(SWF)

Movie S3 **Peripheral deceleration illusion.** Ovals drift from left to right across the screen. Inside each oval is an internal gradient that moves in the direction opposite to the motion of the oval. When viewed foveally, observers can separate the ovals and internal grating; when viewed peripherally, the speed of the ovals is determined by the internal motion, yet it is difficult to see the motion of the internal gradient.
(SWF)

Movie S4 **Rotating reversals illusion.** A ring of ovals moves counter-clockwise. Within each oval is an internal gradient that moves clockwise. When viewed foveally, the ring appears to rotate counter-clockwise, but when viewed peripherally, the ring appears to rotate clockwise. When the background is near the same luminance as the white or black of the internal gradient, the ring appears jumbled when viewed peripherally.
(SWF)

Movie S5 **Disappearing squares illusion.** A) A 16×12 array of Kanizsa pacmen rotate in opposite directions so as to continually assemble/disassemble arrays of Kanizsa squares. As the rotation rate increases, the peripheral range over which the illusory squares can be seen decreases.
(SWF)
